# Spectral Discrete Probability Density Function of Measured Wind Turbine Noise in the Far Field

**DOI:** 10.3389/fpubh.2015.00052

**Published:** 2015-04-07

**Authors:** Payam Ashtiani, Adelaide Denison

**Affiliations:** ^1^Aercoustics Engineering Limited, Toronto, ON, Canada

**Keywords:** wind turbines, infrasound, low-frequency noise, health, spectral, spectrum, probability density, sound pressure level

## Abstract

Of interest is the spectral character of wind turbine noise at typical residential set-back distances. In this paper, a spectral statistical analysis has been applied to immission measurements conducted at three locations. This method provides discrete probability density functions for the Turbine ONLY component of the measured noise. This analysis is completed for one-third octave sound levels, at integer wind speeds, and is compared to existing metrics for measuring acoustic comfort as well as previous discussions on low-frequency noise sources.

## Introduction

Post-construction noise monitoring of wind turbine facilities is becoming more common. Many regulatory agencies are now stipulating long-term measurements of wind turbine noise at residential dwellings post-construction of new wind facilities, as well as in response to community complaints of wind turbine noise. Many jurisdictions have different methodologies for measuring wind turbine noise, and assessing that noise in the determination of compliance.

The Ministry of the Environment and Climate Change (“MOECC”) in Ontario currently stipulates that wind turbine facilities must meet specified noise levels in the surrounding community. As per the MOECC protocol (1), post-construction long-term, unattended measurements are conducted near worst-case receptors (selected based on highest predicted level and predominant down-wind location). Measurements are conducted for both Turbine ON and Turbine OFF operational cases, and the average L_Aeq_ for Turbine OFF is subtracted from the average L_Aeq_ for Turbine ON to determine the average Turbine ONLY component of the measured noise level. This level is then compared to specified limits to determine compliance.

In this paper, a spectral statistical analysis has been conducted on data collected as per the MOECC protocol. This analysis provides a discrete probability density function for measured Turbine ON and Turbine OFF levels, and is then used to infer a discrete probability density function for the Turbine ONLY component. This analysis is done for one-third octave data separated by integer ground-level wind speeds. This inferred Turbine ONLY probability density function allows for detailed analysis of the spectral character of the turbine noise component.

The resulting spectral probability density functions for the Turbine ONLY component provide valuable insight into how often the wind farm operates under certain noise conditions. The probability distribution can provide insight into how often a wind turbine noise is above a certain noise level, or the frequency with which a possible tone is present. This analysis method can also provide clarity when analyzing contaminated data by highlighting discrepancies in background data and is more resilient to contaminating noise sources – such as insect noise.

In this paper, this method has been applied to noise measurements conducted at three different locations at one wind farm. The spectral probability distribution provides insight into data quality and signal-to-noise ratios. The resulting spectral shapes have been compared to existing metrics for acoustic comfort and previous dialog surrounding low-frequency noise (“LFN”) sources.

## Methodology Overview

All receptor-based measurements analyzed for this paper were collected as per the Ontario MOECC’s “Compliance Protocol for Wind Turbine Noise” (1). In accordance with the protocol, the microphone was placed at a height of 4.5 m, and a weather station was located at the same location at a height of 10 m. Sound and weather data were collected simultaneously over 1-min intervals, with sound pressure levels recorded as third-octave L_eq_ levels. Weather data logged included wind speed, wind direction, humidity, temperature, pressure, and precipitation. Sound data collected were sorted into integer wind bins based on the average wind speed measured.

Data collected for each measurement location met the MOECC’s requirements for sample size, with at least 120 data points per wind bin for turbine ON and 60 data points per wind bin for Turbine OFF. For the Turbine OFF (background) component, all turbines in the immediate vicinity of the measurement location were parked such that the predicted level from the wind farm fell by 10 dBA or more – typically to around 30 dBA. Turbine operational data were supplied by the wind farm and cross-referenced to ensure that all turbines were operating during Turbine ON periods, and all relevant turbines were off during background measurements.

### Data filtering

Measurements were only conducted at night (10 p.m.–5 a.m.), when ambient levels are lowest, to allow for the best signal-to-noise ratio possible. Data points were eliminated if the maximum or minimum wind speed measured during the interval differed from the average by more than 2 m/s. To filter for extraneous events, data points were excluded if the L_90_ was more than 6 dB less than the L_eq_, or with a L_eq_ >80 dBA. Due to equipment limitations, data points were filtered if there was any precipitation within an hour and if the temperature dropped below −10C.

### Probability density at a given wind speed and frequency

The MOECC’s Compliance Protocol instructs that the average Turbine OFF overall L_Aeq_ be subtracted from the average Turbine ON overall L_Aeq_ for each wind bin. This gives an average “Turbine ONLY” component, which is then compared to broadband limits specified by the MOECC. This paper approaches the same data set from a statistical basis, and evaluates the turbine contribution of the measured sound level in one-third octave bands.

The proposed methodology aims to provide more insight into the character and frequency distribution of the Turbine ONLY contribution at the receptor and starts with generating a probability density function, as first outlined by Ashtiani (2). This method has been expanded upon for this paper to analyze the frequency content of the turbine component.

**Figure 1:**
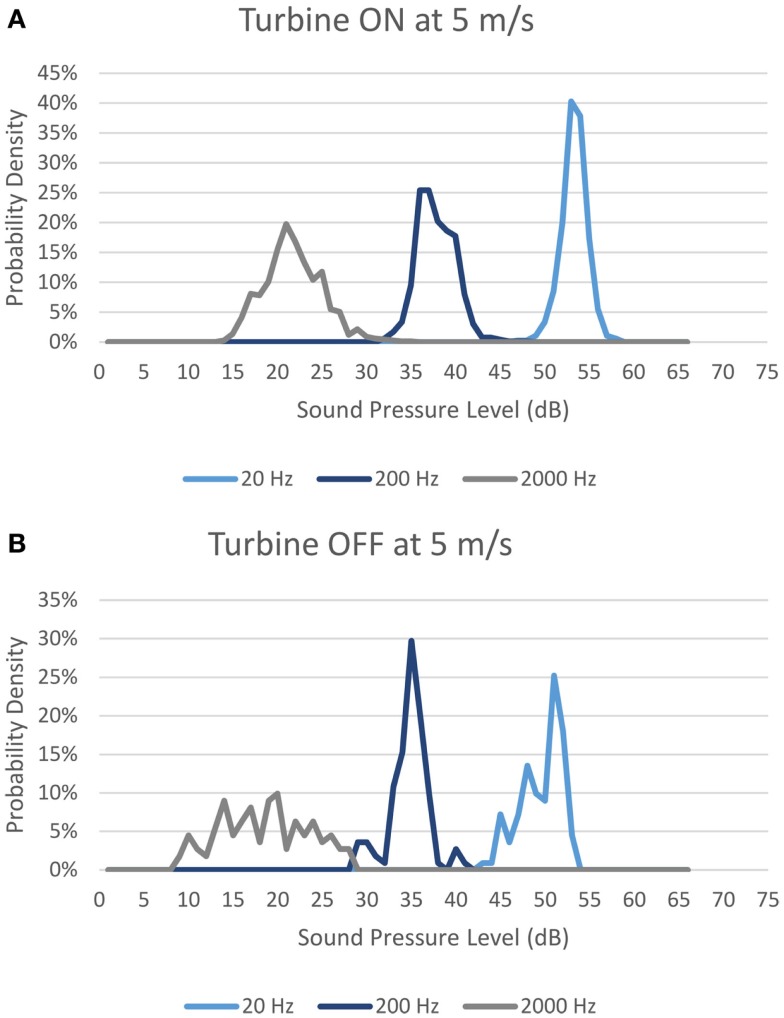
**Probability density for sound pressure level measured with ground-level wind speed of 5 m/s with (A) Turbines ON; and (B) Turbines OFF**.

In every wind bin, for every third octave, the probability density is tabulated. For the example case below, they have been tabulated in 1 dBA increments. Figure 1 shows probability densities for a wind speed of 5 m/s at 20, 200, and 2000 Hz for both Turbine ON and OFF. It should be noted that the example location was measured in the middle of a field at approximately 550 m from the closest turbine.

### Probability density map

**Figure 2:**
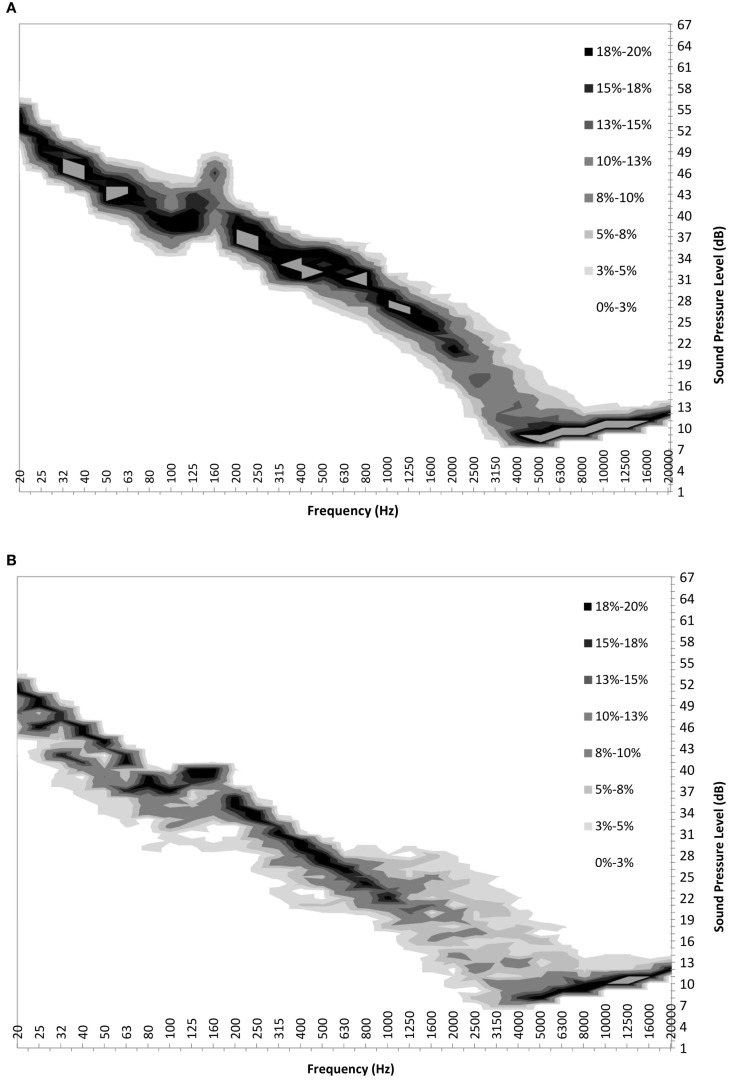
**Probability map for sound pressure level measured with ground-level wind speed of 5 m/s with (A) Turbines ON; and (B) Turbines OFF**.

These probability density functions can be tabulated for each wind bin, at each frequency, for both ON and OFF cases. One-third octave probability density maps can then be generated by graphing the individual probabilities into a “heat map.” Figure 2 shows the maps for the same data set between 20 Hz and 20 kHz inclusive. With this information, one can see the variation in sound level at each frequency both with and without the turbine facility operational.

### Reference sound pressure table

Given that a sufficient sample size is achieved and that the turbine ON/OFF cases represent the same conditions with the exception of the existence of turbine noise, a third probability density map can be devised for the turbine ONLY case. First, a reference table is constructed by which a sound pressure level subtraction is tabulated. In Table S1 in Supplementary Material below, the column headings represent SPL1–SPL2 (logarithmically, of course). For example, if the level at 25 Hz with the turbines ON (SPL1) was 39 dBA and the level at 25 Hz with the turbines OFF (SPL2) was 35, then the contribution of turbines in that scenario, at that frequency, was 37dBA. This example is highlighted in the table.

### Combined probability table

A second table is then constructed that tabulates the probability of each permutation for a given wind speed at a given frequency. For example, a table is constructed for the 5 m/s case at 2000 Hz. The columns would represent turbine ON levels, and the rows would represent turbine OFF levels. The individual entries would represent the probability of both occurring simultaneously based on the probability density maps developed for each case. Table S2 in Supplementary Material shows this constructed table for the 5 m/s wind speed at 2000 Hz.

### Turbine component probability density function

**Figure 3:**
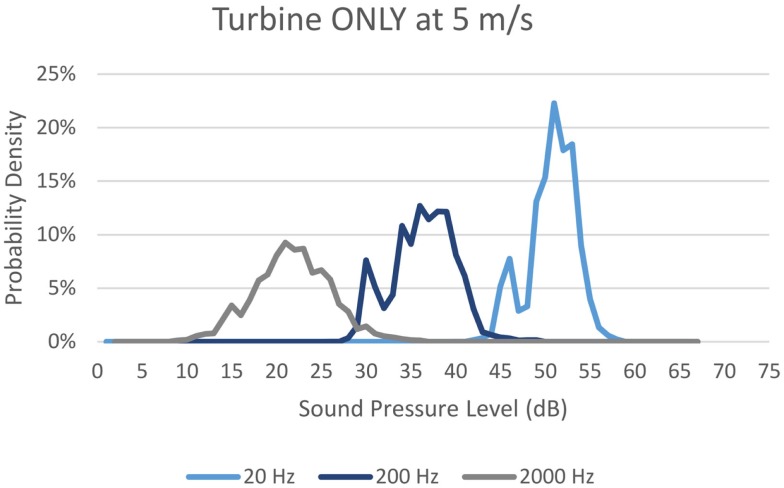
**Probability density for sound pressure level of Turbine ONLY**.

Once both these tables have been constructed for each wind speed and for each frequency, a probability density function can be obtained for the turbine component. Starting at the lowest discretized sound pressure level, one can sum the probabilities of all the instances where the turbine component at a specific frequency results in the sound level of interest. For example, in order to determine the probability of the turbine component being 21 dB at 2000 Hz, one would sum the probabilities of all the instances in reference Table S1 in Supplementary Material where the resulting component is 21 dB. This means that the probability of the turbine component being 21 dB at 2000 Hz is 18%. One can then repeat this process for each discrete sound pressure level. Once complete, the result is a discrete probability density function for the turbine component at that given wind speed and frequency. Figure 3 shows the probability density function for the turbine component at 5 m/s at 20, 200, and 2000 Hz for comparison with Figure 1.

### Turbine component probability density map

**Figure 4:**
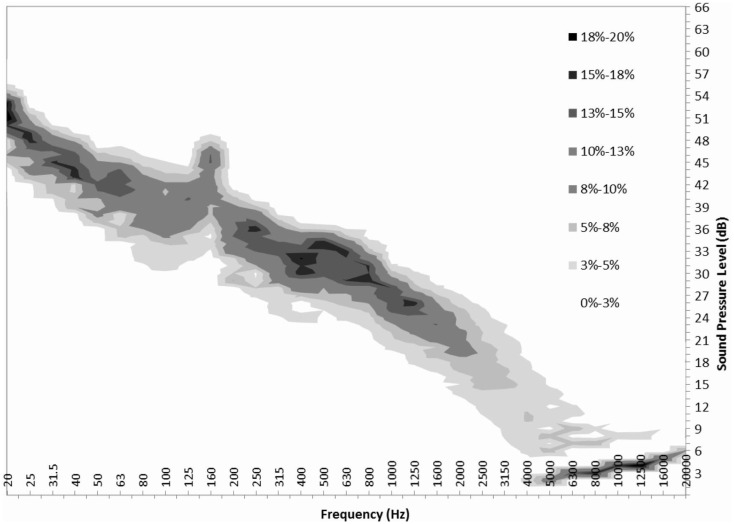
**Probability map for sound pressure level of Turbine ONLY at 5 m/s**.

Once probability density functions are generated for every one-third octave band at a specific wind speed, they can be combined to form a probability density map of the frequency distribution of the turbine only component. A probability density map for 5 m/s can be seen in Figure 4.

With this information, one can get a clearer picture of the frequency distribution of the turbine ONLY component. Using the probability distribution, one can draw conclusions as to how often a turbine might be audible at a certain wind speed, and at which frequencies. Average, 5th and 95th percentile frequency distributions were found for each wind speed at three different measurement locations.

### Comparison to threshold of perception

The turbine component probability density map at each wind speed can be compared to the threshold of perception curve. With insight into the probability of sound pressures occurring at each frequency, one can evaluate how likely the turbine noise is to be perceptible. This comparison is shown in the results Section “Background Probability Maps and Turbine Component Percentiles.”

### Assessment of the slope of the LFN portion

There is also some interest as to whether or not wind turbine noise is a significant source of LFN. Most outdoor LFN guidelines have been developed in order to assess low-frequency tonal noise. The broadband nature of wind turbines noise in the LFN regions does not fit such guidelines. The probability density maps generated in this study were used to compare the spectral levels measured at each measurement location with the preferred noise criteria (PNC) curves (3) as well as the room criterion (RC) Mark II method (4) for designing for acoustic comfort. It is acknowledged that these curves are meant for an indoor sound level. The goal was to determine whether the spectrum shape can be considered disproportionately weighted toward the LFN.

Fiftieth percentile curves from the probability density method were compared with various PNC curves in terms of slope (dB increase per octave band) below 200 Hz. Fiftieth percentile curves were also evaluated with the RC Mark II method for both Turbine ONLY and background for all wind speeds.

### Field measurement campaign

The dataset analyzed for the purposes of this paper includes three measurement locations at the same wind farm in Southern Ontario. The distance from the measurement locations to the nearest turbine for locations A, B, and C are 500, 544, and 485 m, respectively. Measurements were all taken over the same 3-week period in March and April, 2014. Weather conditions included some periods below −10C, but mostly milder temperatures. During the measurement period, there were no crops growing in the fields surrounding the measurement equipment.

## Results

### Turbine component probability maps

**Figure 5:**
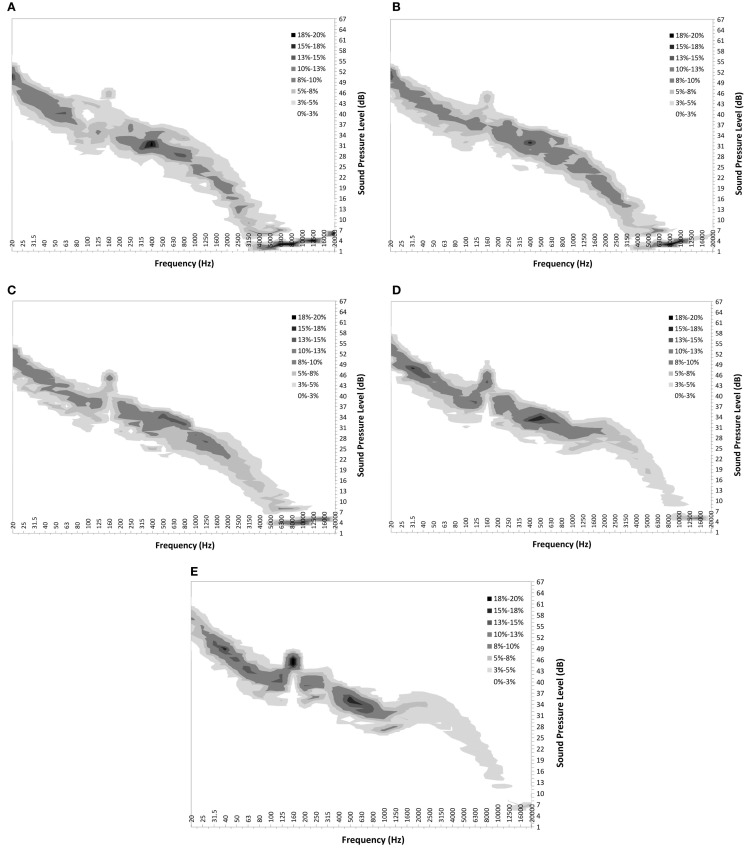
**Turbine ONLY probability distribution maps for measurement location C at wind speeds of 3, 4, 5, 6, and 7 m/s from (A–E) respectively**.

Figure 5 shows graphs of the Turbine ONLY component probability maps for each wind speed from 3 to 7 m/s for measurement location C.

### Background probability maps and turbine component percentiles

**Figure 6:**
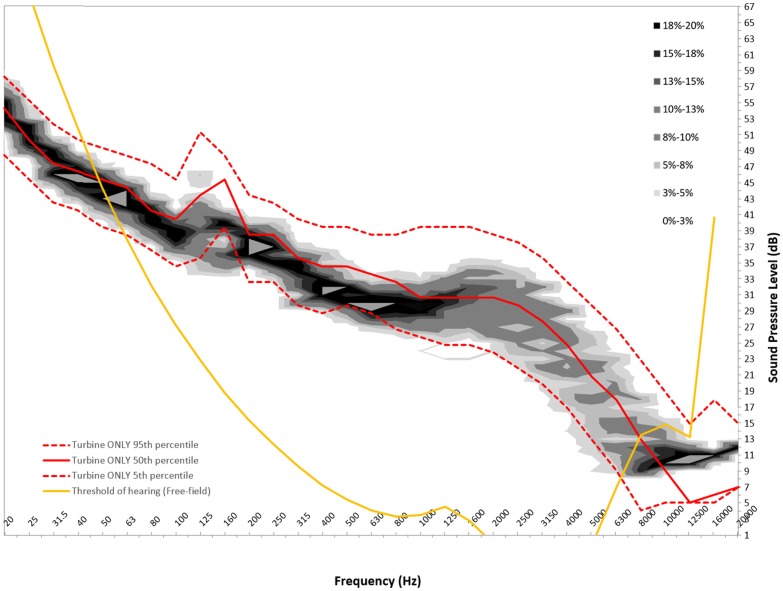
**Six meters per second background probability map with turbine ONLY 5th, 50th, and 95th percentile and 50th percentile reference threshold of hearing curve for measurement location A**.

**Figure 7:**
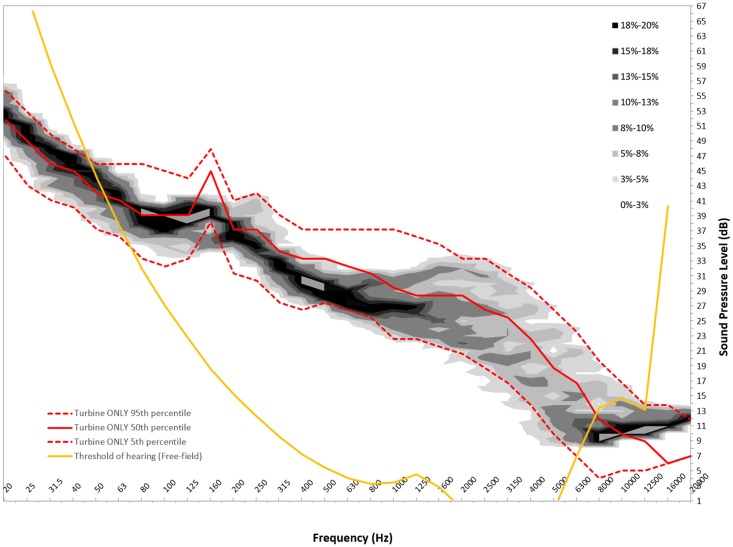
**Six meters per second background probability map with turbine ONLY 5th, 50th, and 95th percentile and 50th percentile reference threshold of hearing curve for measurement location B**.

**Figure 8:**
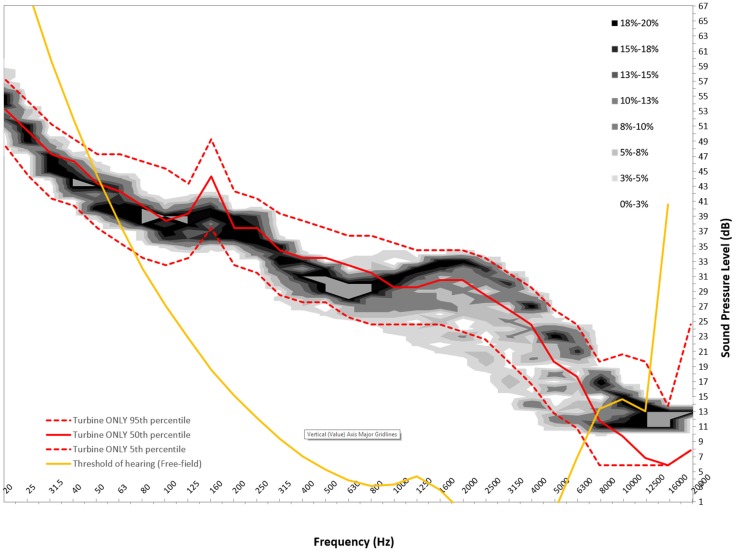
**Six meters per second background probability map with turbine ONLY 5th, 50th, and 95th percentile and 50th percentile reference threshold of hearing curve for measurement location C**.

Figures 6–8 show graphs of the background probability distribution maps for all three measurement locations at 6 m/s overlaid with the 5th, 50th, and 95th percentiles of the turbine ONLY probability distribution along with the 50th percentile threshold of perception curve (5).

### LFN comparison to PNC curves

Since a range of PNC curves are comparable to levels measured at the receptors, an average of the slopes of PNC curves 15–40 below 125 Hz is found to be −7 dB/octave. An average of the slopes of 50th percentiles of Turbine ONLY at all measurement locations and at all wind speeds is an average of −3 dB/octave.

### LFN comparison to RC Mark II rating

Fiftieth percentile curves for both Turbine ONLY and Background sound levels at location C were evaluated using the RC Mark II rating (4) for each wind bin. All spectrums were found to be dominant in the high frequency, with quality assessment indexes (QAI) ranging from 3 dB at low wind speeds to 15 dB at high wind speeds. At each wind speed, the difference in QAI values for Turbine ONLY and Background were 3 dB at the most, with the Background QAI most often higher than that of the Turbine ONLY.

## Discussion

### Limitations of data

This method allows the detection of spectral patterns in the measured noise levels. The statistical aspect of this analysis method also enables interpretation and assessment of the variability of measured sound levels, which is important for wind turbine noise immission measurements. The method is not limited to wind turbine noise, and not inherently limited to any specific distance. As the distance increases, however, the signal-to-noise will deteriorate, and, eventually, the variation in sound level with and without the source present will be indiscernible. It is expected that beyond a distance of 1 km in a typical case, little discernibility would be detectable, except possibly during times when the ground-level wind speeds are very low, and shear conditions cause the sound emission of the turbines to be at or near maximum. This work did not set out to determine the largest distance where a discernable difference was measurable.

The data and analysis presented here is subjected to the following limitations and assumptions:

1. The data sample sizes are assumed to be sufficiently large to adequately describe the population. It is difficult to qualify whether this is the case, as the measurements take place over a duration of about 3 weeks, and the site conditions are always in a certain level of flux. While it is certainly true that the shorter a measurement campaign, the fewer variations in weather conditions would occur, the need for capturing a sufficient number of samples usually means the measurement campaign can take long.
2. As one can glean from the low signal-to-noise ratio in the lower frequencies, it is possible that the measurement system was effected by the self-noise of the wind screen at those low frequencies. Despite using a secondary windscreen to shield from low-frequency pseudo-noise from wind over the microphones, it is not guaranteed that the measurements are not influenced significantly in those frequencies. It is also likely, however, that the ambient noise level is providing the masking noise that causes the poor signal-to-noise. The resulting measured LFN in this work agrees with what was measured by Tachibana et al. (6), with their most protective measurement configuration, and in some cases this set of measurements are even lower those published by Tachibana. Thus, the measurement system is expected to be adequate. Further study in this field is generally needed.

### Low-frequency noise component

There is considerable interest in the low-frequency content sound immission measured from wind turbines. In order to use this statistical method to gain insight into the spectral content, the ISO 389-7 threshold of sound perception for pure tones is plotted over the measured sound contributions from the turbine, as well as the background. For this analysis, the 50th percentiles for the measured level are presented. It should be noted that the threshold curve taken from ISO 389-7 represents the level of a sound at which “a person gives 50% correct detection responses on repeated trials” (5).

From Figures 6–8, we can see that below 50 Hz the measured level contribution from the turbines is at or below the threshold. This is consistent with other studies such as Moller and Pedersen (7), Sondegaard and Madsen (8) and O’Neal (9). For the measurements conducted in this study, the analysis has shown that the measurements have a significant contribution from ambient conditions, and that the signal-to-noise ratio is generally 3 dB or less. Consequently, the measured levels are typically close to ambient conditions, and generally become more audible with increasing frequency. It is important to note that LFN is generally accepted as being sound in the frequency range of 20–200 Hz. Thus, wind turbine noise certainly has perceptible noise levels measurable in the low-frequency range. The highest audibility of the measured levels was in the higher portion of the low-frequency range, where levels are between 2 and 6 dB above ambient conditions. The noise comprises in this case of broadband noise from the turbine, as well as some tonal components around 160 Hz frequency range.

The low-frequency spectrum shape was also compared to other references in order to evaluate whether the broadband component of the measured noise impact from the turbines represents a spectrum that is unbalanced with excess low-frequency content. Due to a general lack of listener tested criterion for broadband LFN, comparison was made to target spectral noise for buildings and interior spaces. PNC curves and RC curves have been developed for designing the interior acoustics of spaces when controlling building noise from HVAC (3, 4). The targets are meant for acoustic comfort and minimizing base building noise complaints.

The slopes of the low-frequency portion of the PNC curves were evaluated and compared to the measured levels from wind turbines. The slopes of the measurements between 25 and 160 Hz averaged a reduction of 3 dB/octave. This is similar to the rate of 4 dB/octave measured by Tachibana (6). Compared to PNC curves of 40 and below, this represents a low slope, with would imply that the shape of the spectrum does not by itself indicate a noise that is unbalanced toward the low-frequency region.

The RC Mark II method was applied to the 50th percentile curves for Turbine ONLY and Background for each wind bin. A QAI was calculated for each. A QAI value over 5 dB indicates a spectrum shape, which a listener is likely to find objectionable. In almost all cases, the QAI was found to be >5 dB, but Turbine ONLY and Background QAI values were very comparable at each wind speed, with the QAI most often higher for the background noise. Further, all spectral profiles were found to be dominant in the high frequency using this method. The standard describes that a noise with this spectral profile would be heard as a hiss, and based on the background levels typically having a higher QAI than Turbine ONLY, it is expected that the high-frequency content is due to ambient wind noise.

It should be noted that the measurements carried out in this study are 1-min energy averaged intervals. Amplitude modulations that may occur during the measurement intervals are not explicitly identified with this methodology, but are included in the energy averaging.

Additionally, it is acknowledged that both the PNC and RC Mark II evaluation methods are meant for indoor sound environments. The high-frequency portion of the spectra will be reduced significantly at indoor locations. This would also therefore reduce the QAI value for both the Turbine ONLY and Background noise levels.

### Mid frequency noise component

Mid frequency noise from about 200 Hz to 2 kHz represents the majority of the impact of wind turbine noise at the immission point. This frequency range is one where the sound from the turbines is expected to be most audible, and under certain conditions result in the highest change in ambient conditions.

Signal-to-noise ratio of the Turbine ONLY component in our study was found as high as 5 dB in the 400–500 Hz range. One should note that this meant the average signal with Turbines ON was 6 dB above the ambient level. Above 1000 Hz, and at high wind speeds, the signal-to-noise begins to deteriorate rapidly as ambient noise increases sharply. The spectrum shape above 2000 Hz is strongly influenced by the ambient condition, and the level of ambient noise at a given measurement location.

The benefit of a spectral statistical analysis is its increased robustness to contaminating noise in select frequencies. A common example of this is cricket or other insect noise. Frequently, when insect noise contaminates the signal, it will drive the overall A-weighted sound level both for the turbine ON and turbine OFF measurements. If one compares the two levels, it is difficult to discern any contribution attributable to the turbines. A spectral analysis will be able to discount the frequencies where insect noise was present, and provide a turbine noise component based on the remaining frequencies where insect noise did not dominate the measured levels.

## Conclusion

A spectral statistical analysis is presented for measured noise levels near wind farms (at typical residential dwelling setbacks of 500 m or greater – this approach may be applicable at distances >1 km, but only under certain meteorological conditions). The method allows for inferring the discrete probability density function of noise components in the signal attributable to wind turbines, at integer wind speeds. The method also allows for the computation of cumulative percentages of time that the noise impact from the wind farm meets a specific criterion. It provides a greater insight as to the frequency of occurrence of noises that are of interest, or allows the total amount of time that a wind farm may be in a non-compliance state to be quantified. It is hoped that this kind of analysis will provide insight into the variability of the ambient noise environment, combined with the variability of the sound impact of the turbines at typical distances. This approach would allow regulators to prescribe noise limits that are statistically defined. For example, a noise limit of 45 dBA that shall not be exceeded 95% of the time. This approach would also discourage the use of “cherry-picked” data during single measurement events to characterize the noise immission behavior of a given wind turbine facility.

## Conflict of Interest Statement

In terms of competing interests (financial and non-financial), the authors work for a consulting firm and have worked with wind power companies. The authors are actively working in the field of wind turbines as acoustical engineers. Although we make this disclosure, we wish to reiterate that as independent scientific professionals, our views and research are not influenced by these contractual obligations.

## Supplementary Material

The Supplementary Material for this article can be found online at http://journal.frontiersin.org/article/10.3389/fpubh.2015.00052
