# Supplementary material for: Spectral Discrete Probability Density Function of Measured Wind Turbine Noise in the Far Field
Source: Front Public Health. 2015 Apr 7;3:52. doi: 10.3389/fpubh.2015.00052 (PMC4387936; doi:10.3389/fpubh.2015.00052)
Supplement: Supplementary file 1 [file Table_1.PDF]

Table 1: Reference SPL Contribution (SPL1-SPL2)

SPL 1 (columns) / SPL 2 (rows)

dBA	66	65	64	63	62	61	60	59	58	57	56	55	54	53	52	51	50	49	48	47	46	45	44	43	42	41	40	39	38	37	36	35	34	33	32	31	30	29	28	27	26	25	24	23	22	21	20	19	18	17	16	15	14	13	12	11	10	9	8	7	6	5	4	3	2	1
66
65	59
64	62	58
63	63	61	57
62	64	62	60	56
61	64	63	61	59	55
60	65	63	62	60	58	54
59	65	64	62	61	59	57	53
58	65	64	63	61	60	58	56	52
57	65	64	63	62	60	59	57	55	51
56	66	64	63	62	61	59	58	56	54	50
55	66	65	63	62	61	60	58	57	55	53	49
54	66	65	64	62	61	60	59	57	56	54	52	48
53	66	65	64	63	61	60	59	58	56	55	53	51	47
52	66	65	64	63	62	60	59	58	57	55	54	52	50	46
51	66	65	64	63	62	61	59	58	57	56	54	53	51	49	45
50	66	65	64	63	62	61	60	58	57	56	55	53	52	50	48	44
49	66	65	64	63	62	61	60	59	57	56	55	54	52	51	49	47	43
48	66	65	64	63	62	61	60	59	58	56	55	54	53	51	50	48	46	42
47	66	65	64	63	62	61	60	59	58	57	55	54	53	52	50	49	47	45	41
46	66	65	64	63	62	61	60	59	58	57	56	54	53	52	51	49	48	46	44	40
45	66	65	64	63	62	61	60	59	58	57	56	55	53	52	51	50	48	47	45	43	39
44	66	65	64	63	62	61	60	59	58	57	56	55	54	52	51	50	49	47	46	44	42	38
43	66	65	64	63	62	61	60	59	58	57	56	55	54	53	51	50	49	48	46	45	43	41	37
42	66	65	64	63	62	61	60	59	58	57	56	55	54	53	52	50	49	48	47	45	44	42	40	36
41	66	65	64	63	62	61	60	59	58	57	56	55	54	53	52	51	49	48	47	46	44	43	41	39	35
40	66	65	64	63	62	61	60	59	58	57	56	55	54	53	52	51	50	48	47	46	45	43	42	40	38	34
39	66	65	64	63	62	61	60	59	58	57	56	55	54	53	52	51	50	49	47	46	45	44	42	41	39	37	33
38	66	65	64	63	62	61	60	59	58	57	56	55	54	53	52	51	50	49	48	46	45	44	43	41	40	38	36	32
37	66	65	64	63	62	61	60	59	58	57	56	55	54	53	52	51	50	49	48	47	45	44	43	42	40	39	37	35	31
36	66	65	64	63	62	61	60	59	58	57	56	55	54	53	52	51	50	49	48	47	46	44	43	42	41	39	38	36	34	30
35	66	65	64	63	62	61	60	59	58	57	56	55	54	53	52	51	50	49	48	47	46	45	43	42	41	40	38	37	35	33	29
34	66	65	64	63	62	61	60	59	58	57	56	55	54	53	52	51	50	49	48	47	46	45	44	42	41	40	39	37	36	34	32	28
33	66	65	64	63	62	61	60	59	58	57	56	55	54	53	52	51	50	49	48	47	46	45	44	43	41	40	39	38	36	35	33	31	27
32	66	65	64	63	62	61	60	59	58	57	56	55	54	53	52	51	50	49	48	47	46	45	44	43	42	40	39	38	37	35	34	32	30	26
31	66	65	64	63	62	61	60	59	58	57	56	55	54	53	52	51	50	49	48	47	46	45	44	43	42	41	39	38	37	36	34	33	31	29	25
30	66	65	64	63	62	61	60	59	58	57	56	55	54	53	52	51	50	49	48	47	46	45	44	43	42	41	40	38	37	36	35	33	32	30	28	24
29	66	65	64	63	62	61	60	59	58	57	56	55	54	53	52	51	50	49	48	47	46	45	44	43	42	41	40	39	37	36	35	34	32	31	29	27	23
28	66	65	64	63	62	61	60	59	58	57	56	55	54	53	52	51	50	49	48	47	46	45	44	43	42	41	40	39	38	36	35	34	33	31	30	28	26	22
27	66	65	64	63	62	61	60	59	58	57	56	55	54	53	52	51	50	49	48	47	46	45	44	43	42	41	40	39	38	37	35	34	33	32	30	29	27	25	21
26	66	65	64	63	62	61	60	59	58	57	56	55	54	53	52	51	50	49	48	47	46	45	44	43	42	41	40	39	38	37	36	34	33	32	31	29	28	26	24	20
25	66	65	64	63	62	61	60	59	58	57	56	55	54	53	52	51	50	49	48	47	46	45	44	43	42	41	40	39	38	37	36	35	33	32	31	30	28	27	25	23	19
24	66	65	64	63	62	61	60	59	58	57	56	55	54	53	52	51	50	49	48	47	46	45	44	43	42	41	40	39	38	37	36	35	34	32	31	30	29	27	26	24	22	18
23	66	65	64	63	62	61	60	59	58	57	56	55	54	53	52	51	50	49	48	47	46	45	44	43	42	41	40	39	38	37	36	35	34	33	31	30	29	28	26	25	23	21	17
22	66	65	64	63	62	61	60	59	58	57	56	55	54	53	52	51	50	49	48	47	46	45	44	43	42	41	40	39	38	37	36	35	34	33	32	30	29	28	27	25	24	22	20	16
21	66	65	64	63	62	61	60	59	58	57	56	55	54	53	52	51	50	49	48	47	46	45	44	43	42	41	40	39	38	37	36	35	34	33	32	31	29	28	27	26	24	23	21	19	15
20	66	65	64	63	62	61	60	59	58	57	56	55	54	53	52	51	50	49	48	47	46	45	44	43	42	41	40	39	38	37	36	35	34	33	32	31	30	28	27	26	25	23	22	20	18	14
19	66	65	64	63	62	61	60	59	58	57	56	55	54	53	52	51	50	49	48	47	46	45	44	43	42	41	40	39	38	37	36	35	34	33	32	31	30	29	27	26	25	24	22	21	19	17	13
18	66	65	64	63	62	61	60	59	58	57	56	55	54	53	52	51	50	49	48	47	46	45	44	43	42	41	40	39	38	37	36	35	34	33	32	31	30	29	28	26	25	24	23	21	20	18	16	12
17	66	65	64	63	62	61	60	59	58	57	56	55	54	53	52	51	50	49	48	47	46	45	44	43	42	41	40	39	38	37	36	35	34	33	32	31	30	29	28	27	25	24	23	22	20	19	17	15	11
16	66	65	64	63	62	61	60	59	58	57	56	55	54	53	52	51	50	49	48	47	46	45	44	43	42	41	40	39	38	37	36	35	34	33	32	31	30	29	28	27	26	24	23	22	21	19	18	16	14	10
15	66	65	64	63	62	61	60	59	58	57	56	55	54	53	52	51	50	49	48	47	46	45	44	43	42	41	40	39	38	37	36	35	34	33	32	31	30	29	28	27	26	25	23	22	21	20	18	17	15	13	9
14	66	65	64	63	62	61	60	59	58	57	56	55	54	53	52	51	50	49	48	47	46	45	44	43	42	41	40	39	38	37	36	35	34	33	32	31	30	29	28	27	26	25	24	22	21	20	19	17	16	14	12	8
13	66	65	64	63	62	61	60	59	58	57	56	55	54	53	52	51	50	49	48	47	46	45	44	43	42	41	40	39	38	37	36	35	34	33	32	31	30	29	28	27	26	25	24	23	21	20	19	18	16	15	13	11	7
12	66	65	64	63	62	61	60	59	58	57	56	55	54	53	52	51	50	49	48	47	46	45	44	43	42	41	40	39	38	37	36	35	34	33	32	31	30	29	28	27	26	25	24	23	22	20	19	18	17	15	14	12	10	6
11	66	65	64	63	62	61	60	59	58	57	56	55	54	53	52	51	50	49	48	47	46	45	44	43	42	41	40	39	38	37	36	35	34	33	32	31	30	29	28	27	26	25	24	23	22	21	19	18	17	16	14	13	11	9	5
10	66	65	64	63	62	61	60	59	58	57	56	55	54	53	52	51	50	49	48	47	46	45	44	43	42	41	40	39	38	37	36	35	34	33	32	31	30	29	28	27	26	25	24	23	22	21	20	18	17	16	15	13	12	10	8	4
9	66	65	64	63	62	61	60	59	58	57	56	55	54	53	52	51	50	49	48	47	46	45	44	43	42	41	40	39	38	37	36	35	34	33	32	31	30	29	28	27	26	25	24	23	22	21	20	19	17	16	15	14	12	11	9	7	3
8	66	65	64	63	62	61	60	59	58	57	56	55	54	53	52	51	50	49	48	47	46	45	44	43	42	41	40	39	38	37	36	35	34	33	32	31	30	29	28	27	26	25	24	23	22	21	20	19	18	16	15	14	13	11	10	8	6	2
7	66	65	64	63	62	61	60	59	58	57	56	55	54	53	52	51	50	49	48	47	46	45	44	43	42	41	40	39	38	37	36	35	34	33	32	31	30	29	28	27	26	25	24	23	22	21	20	19	18	17	15	14	13	12	10	9	7	5	1
6	66	65	64	63	62	61	60	59	58	57	56	55	54	53	52	51	50	49	48	47	46	45	44	43	42	41	40	39	38	37	36	35	34	33	32	31	30	29	28	27	26	25	24	23	22	21	20	19	18	17	16	14	13	12	11	9	8	6	4	0
5	66	65	64	63	62	61	60	59	58	57	56	55	54	53	52	51	50	49	48	47	46	45	44	43	42	41	40	39	38	37	36	35	34	33	32	31	30	29	28	27	26	25	24	23	22	21	20	19	18	17	16	15	13	12	11	10	8	7	5	3	-1
4	66	65	64	63	62	61	60	59	58	57	56	55	54	53	52	51	50	49	48	47	46	45	44	43	42	41	40	39	38	37	36	35	34	33	32	31	30	29	28	27	26	25	24	23	22	21	20	19	18	17	16	15	14	12	11	10	9	7	6	4	2	-2
3	66	65	64	63	62	61	60	59	58	57	56	55	54	53	52	51	50	49	48	47	46	45	44	43	42	41	40	39	38	37	36	35	34	33	32	31	30	29	28	27	26	25	24	23	22	21	20	19	18	17	16	15	14	13	11	10	9	8	6	5	3	1	-3
2	66	65	64	63	62	61	60	59	58	57	56	55	54	53	52	51	50	49	48	47	46	45	44	43	42	41	40	39	38	37	36	35	34	33	32	31	30	29	28	27	26	25	24	23	22	21	20	19	18	17	16	15	14	13	12	10	9	8	7	5	4	2	0	-4
1	66	65	64	63	62	61	60	59	58	57	56	55	54	53	52	51	50	49	48	47	46	45	44	43	42	41	40	39	38	37	36	35	34	33	32	31	30	29	28	27	26	25	24	23	22	21	20	19	18	17	16	15	14	13	12	11	9	8	7	6	4	3	1	-1	-5
